# Clinical Manifestations and Outcomes of West Nile Virus Infection

**DOI:** 10.3390/v6020606

**Published:** 2014-02-06

**Authors:** James J. Sejvar

**Affiliations:** National Center for Emerging and Zoonotic Infectious Diseases, Centers for Disease Control and Prevention, Atlanta, GA 30333, USA; E-Mail: zea3@cdc.gov; Tel.: +1-404-639-4657; Fax: +1-404-639-3838

**Keywords:** West Nile virus, meningitis, encephalitis, poliomyelitis, outcomes

## Abstract

Since the emergence of West Nile virus (WNV) in North America in 1999, understanding of the clinical features, spectrum of illness and eventual functional outcomes of human illness has increased tremendously. Most human infections with WNV remain clinically silent. Among those persons developing symptomatic illness, most develop a self-limited febrile illness. More severe illness with WNV (West Nile neuroinvasive disease, WNND) is manifested as meningitis, encephalitis or an acute anterior (polio) myelitis. These manifestations are generally more prevalent in older persons or those with immunosuppression. In the future, a more thorough understanding of the long-term physical, cognitive and functional outcomes of persons recovering from WNV illness will be important in understanding the overall illness burden.

## 1. Introduction

In the decade since the emergence of West Nile virus (WNV) in North America, our collective understanding of the clinical manifestations, prognostic factors and short- and long-term outcomes of illness has increased dramatically. Previous historic outbreaks of WNV in sub-Saharan Africa and the Middle East had typically been characterized as mild, self-limited febrile illness with few or no sequelae [1]. More recently, beginning in the 1990s, more cases of neurologic illness, particularly “neuroinvasive” disease, in which the virus directly infects the nervous system, began to be recognized [1,2]. However, the ongoing outbreak of WNV in North America has greatly expanded our understanding of the spectrum of illness associated with WNV infection in humans, and a number of previously under-recognized syndromes have been characterized [3,4,5,6]. 

It is generally estimated that about 80% of persons infected with WNV remain asymptomatic. Of those who develop symptoms, the vast majority develop an acute, systemic febrile illness (“West Nile fever”, WNF). Data suggest that less than 1% of infected persons develop neurologic illness, which is primarily attributed to neuroinvasive disease, in which the virus breaches the intrathecal space and produces infection of central nervous system (CNS) structures. Neuroinvasive disease includes aseptic meningitis (“West Nile meningitis”, WNM), encephalitis (“West Nile encephalitis”, WNE) or an acute poliomyelitis-like syndrome (“West Nile poliomyelitis”, WNP) [1,2]. WNM involves infection of the meninges (the outer covering of the brain and spinal cord) and makes up the largest percentage of neuroinvasive disease cases in younger age groups. WNE involves viral infection of the brain parenchyma itself and is more typically manifested in older persons or individuals with compromised immune systems. WNP results from viral infection of the anterior horn cells of the spinal cord, leading to acute flaccid limb weakness. When discussing the various manifestations of WNV infection, it is important to keep in mind that the clinical picture may not always be as clear-cut as delineated above, and sometimes, the clinical features of WNF, WNM and WNE may overlap. For example, patients may develop an altered mental status, due to severe systemic illness, without true histopathologic or radiologic evidence of cerebral inflammation or “encephalitis” in the pathophysiologic sense. Similarly, patients presenting with fever, headache and “neck stiffness” may not undergo lumbar puncture to demonstrate pleocytosis, and consequently, a diagnosis of WN “meningitis” may not be reported. Despite these limitations, the clinical syndrome in most persons with WNV illness can be diagnosed based upon clinical grounds.

## 2. Clinical Syndromes Associated with WNV Infection

### 2.1. West Nile Fever (WNF)

WNF is the predominant clinical syndrome seen in most infected persons. All ages may be affected, but data suggest that the proportion of WNF may be higher among younger individuals [5,7,8,9]. Following an incubation period of approximately 2–14 days, infected persons typically experience the abrupt onset of fever, headache, fatigue and myalgias. Gastrointestinal complaints, including nausea and vomiting, have been frequently described and may lead to dehydration.

**Figure 1:**
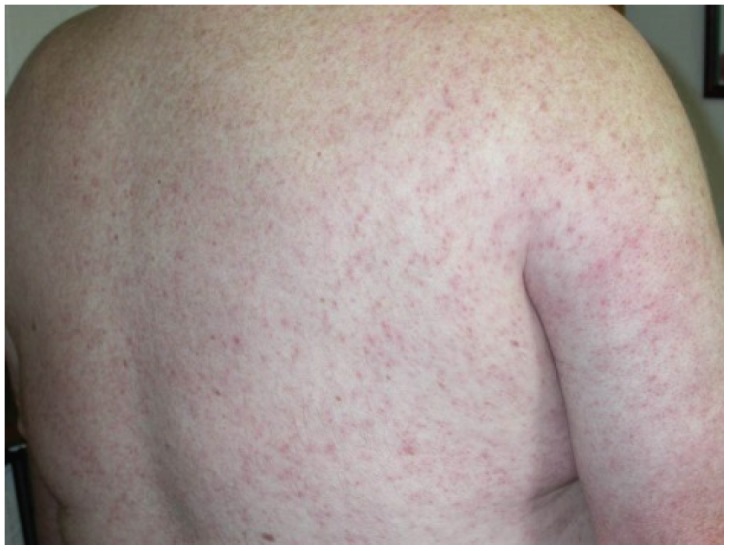
Diffuse maculopapular rash associated with West Nile virus infection.

WNF may sometimes be associated with a rash, which tends to be morbilliform, maculopapular and non-pruritic and predominates over the torso and extremities, sparing the palms and soles [10,11,12,13]. (Figure 1). The rash may be transient, lasting less than 24 h in some persons. Interestingly, this rash appears to be more frequently seen in WNF than in more severe illness manifestations (WNM or WNE) [10]. In addition, rash is more frequently observed among younger persons than among older persons [10]. These findings raise the question as to whether the presence of a rash correlates with host immune or cytokine response to infection.

Although elderly persons with WNF may experience adverse outcomes and have a higher mortality rate than younger symptomatic persons [4,8], most patients experience complete recovery. Some otherwise healthy persons, however, may continue to experience persistent fatigue, headaches and difficulties concentrating for days or weeks following infection [14]. In particular, profound fatigue, sometimes interfering with work or school activities, may last for months among persons recovering from WNF [15]. Deaths among persons with WNF occur primarily among older persons and the immunocompromised population and are frequently attributable to cardiopulmonary complications [16].

### 2.2. West Nile Neuroinvasive Disease

#### 2.2.1. West Nile Meningitis (WNM)

WNM is essentially indistinguishable from other viral meningitides (or “aseptic meningitis”). Persons developing WNM experience the abrupt onset of fever and headache and demonstrate meningeal signs, including nuchal rigidity, Kernig’s and/or Brudzinski’s signs and photophobia or phonophobia. The associated headache may be severe, requiring hospitalization for pain control; associated gastrointestinal symptoms, such as nausea, vomiting and diarrhea, may result in dehydration, exacerbating head pain and systemic symptoms [15]. WNM is generally associated with a favorable outcome, though, similar to WNF, some patients experience persistent headache, fatigue and myalgias [3,15]. 

Cerebrospinal fluid (CSF) examination is characterized by a modest pleocytosis (elevation of white blood cells), generally less than 500 cells/mm^3^. While this pleocytosis is usually lymphocytic, which is typical of viral meningitis, CSF obtained soon after the onset of symptoms may show a neutrophilic predominance [17,18]. The presence of plasma cells has been suggested to be indicative of WN virus infection [19]; however, this finding requires further substantiation.

#### 2.2.2. West Nile Encephalitis (WNE)

WNE may range in severity from a mild, self-limited confusional state to severe encephalopathy, coma and death. This manifestation is more commonly seen in older individuals, particularly over the age of 55, as well as immunocompromised persons [20,21]. Several neurological syndromes, primarily extrapyramidal disorders, have been observed in patients with WNE [3,5,6,22] and, in the setting of known virus circulation, may be very suggestive of WNV as the etiological agent for the encephalitis [3]. Patients with WNE frequently develop a coarse bilateral tremor, particularly in the upper extremities. The tremor tends to be postural and may have a kinetic component [3,4,6,22]. Myoclonus, predominantly of the upper extremities and facial muscles, may occur and may be present during sleep. Features of parkinsonism, including hypomimia, bradykinesia and postural instability, may be seen and can be associated with falls and functional difficulties [3,23]. Cerebellar ataxia, with associated truncal instability and gait disturbance, leading to falls, has been described [6,22,24]. These abnormal movements usually follow the onset of mental status changes. Typically, these movement disorders resolve over time; however, tremor and parkinsonism may persist in patients recovering from severe encephalitis [3,5]. 

The development of these movement disorders in WNE is due to specific neurotropism of WNV for extrapyramidal structures; there is frequent involvement of the brainstem (particularly, the medulla and pons), the deep gray matter nuclei, particularly the substantia nigra of the basal ganglia and the thalami, and the cerebellum [25,26,27]. This clinico-pathologic correlation may be extended to the neuroimaging abnormalities seen in West Nile encephalitis. 

**Figure 2:**
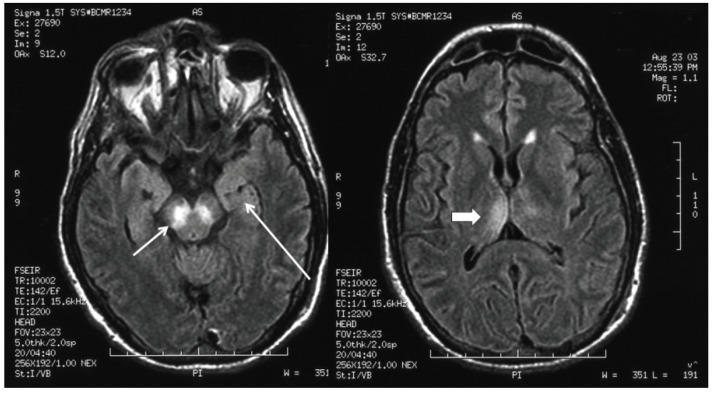
Fluid-attenuated inversion recovery magnetic resonance imaging sequence of the brain in a patient with West Nile virus encephalitis with associated parkinsonism and tremor, displaying signal abnormality in the substantia nigra (short arrow), the mesial temporal lobe (long arrow) and right posterior thalamus (thick arrow).

The estimated proportion of patients with WNE who demonstrate abnormal findings in brain magnetic resonance imaging (MRI) varies considerably between studies; however, MRI findings are not ubiquitous, and even in cases of severe WNE, the MRI may be normal; or abnormal findings may not be apparent until several weeks after the onset of illness [28,29,30]. The most characteristic MRI findings in patients with WNE are bilateral signal abnormalities in the basal ganglia and thalami on T2- , fluid-attenuated inversion recovery (FLAIR) and diffusion-weighted image sequences, indicating the viral neurotropism for these deep gray structures (Figure 2). These MRI findings, which may be seen in other flaviviral encephalitides, including encephalitis, due to Japanese encephalitis virus and St. Louis encephalitis virus, may be indicative, but not diagnostic for, WNE. Electroencephalographic (EEG) abnormalities may be present in the form of generalized slowing, frequently anteriorly or temporally predominant, and triphasic sharp waves [31,32]. These EEG abnormalities, however, are also nonspecific. Overt seizures appear to be relatively uncommon with WNE and are estimated to occur in 3%–6% of patients [33]. Similarly, increased intracranial pressure and cerebral edema appear to be uncommon in WNE. CSF abnormalities in patients with WNE are essentially the same as those seen in WNM, characterized by moderate lymphocytic pleocytosis, elevated protein and normal glucose. One large study suggested that the mean CSF white blood cell count in patients with WNE was 227 cells/mm^3^ (median, 90 cells/mm^3^) [34].

Neuropsychiatric symptoms, including depression, anxiety and apathy, have been reported among patients recovering from WNE [35,36]. Fatality rates from WNE have ranged from between 10% and 30%, with mortality higher among older persons and immunocompromised individuals [1,2,37]. Of note, the severity of initial encephalitic illness does not necessarily predict ultimate outcome, as some individuals developing severe WNE have been observed to go on to have full recovery [3].

#### 2.2.3. West Nile Poliomyelitis (WNP) and Other Forms of Acute Flaccid Paralysis (AFP)

Acute and abrupt onset of limb weakness may be seen in WNV infection; in most cases, this limb paresis (partial weakness) or paralysis (complete loss of muscle power) is due to viral involvement of the lower motor neurons of the spinal cord (anterior horn cells), resulting in anterior (polio) myelitis [18,38,39,40,41,42]. This syndrome, typically associated with poliovirus, may be caused by a number of other viruses [43,44]. The clinical features of WNP are characteristic and generally dramatic, allowing for differentiation from the characteristic diffuse “muscle weakness” described by many persons with severe fatigue associated with WNV infection (Table 1). WNP generally develops soon after illness onset, usually within the first 24 to 48 h. Limb weakness generally develops rapidly and may be abrupt, occasionally raising clinical concern about stroke [45,46]. The weakness is usually asymmetric and often results in monoplegia (weakness of one limb). Patients with severe and extensive spinal cord involvement develop a more symmetric dense quadriplegia. Central facial weakness, frequently bilateral, can also be seen [38]. Sensory loss or numbness is generally absent, though some patients experience intense pain in the affected limbs just before or during the onset of weakness, and this limb pain may be persistent [18].

**Table 1:** Clinical and electrodiagnostic features of different types of weakness associated with West Nile virus infection.

Characteristic	West Nile Poliomyelitis	Guillain–Barré Syndrome	Fatigue-Related “Muscle Weakness”
*Timing of onset*	Acute phase of infection	One to eight weeks following acute infection	Acute infection
*Fever and leukocytosis*	Present	Absent	Present
*Weakness distribution*	Asymmetric; occasional monoplegia	Generally symmetric; proximal and distal muscles	Generalized, subjective, but neurologic examination normal
*Sensory symptoms*	Absence of numbness, paresthesias or sensory loss; pain often present	Painful distal paresthesias and sensory loss	Generally absent
*Bowel/bladder involvement*	Often present	Rare	Not present
*Concurrent encephalopathy*	Often present	Generally absent	May be seen with fever, meningitis or encephalitis
*CSF Profile*	Pleocytosis and elevated protein	No pleocytosis; elevated protein (albuminocytologic dissociation)	Pleocytosis and elevated protein in the setting of meningitis/encephalitis

The most severe manifestation of WNP is the involvement of respiratory muscle innervation, leading to diaphragmatic and intercostal muscle paralysis and resulting in neuromuscular respiratory failure, requiring emergency endotracheal intubation [18,47]. Involvement of the lower brainstem, including the motor nuclei of the vagus and glossopharyngeal nerves, is similar to that seen in poliovirus infection and appears to be the underlying pathophysiologic basis for this manifestation [33,48]. Respiratory involvement in WNP is associated with high morbidity and mortality, and among survivors, prolonged ventilatory support lasting months may be required [18]; unfortunately, in some cases, patients are unable to be weaned from mechanical ventilation, and the withdrawal of ventilator support leads to death. Patients who develop bulbar findings, such as dysarthria, dysphagia or loss of gag reflex, are at greater risk for respiratory failure and should be monitored closely.

**Figure 3:**
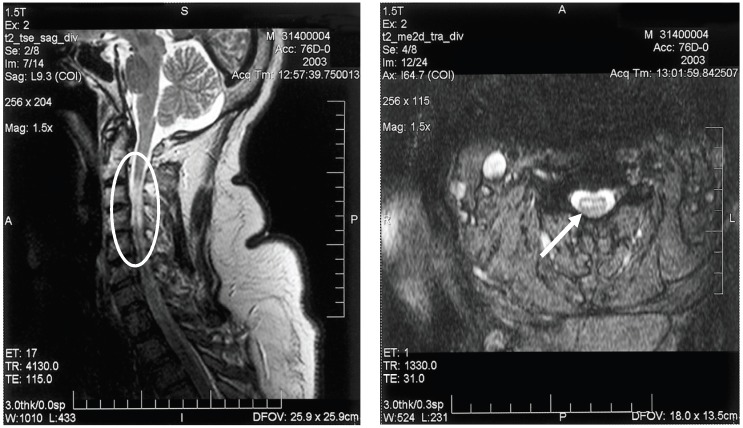
Sagittal (**A**) and axial (**B**) T2-weighted magnetic resonance imaging of the cervical spinal cord of a patient with bilateral upper extremity paralysis and respiratory failure from West Nile poliomyelitis, displaying the increased signal in the anterior spinal cord (circle and arrow).

Electrodiagnostic studies (electromyography/nerve conduction studies) will display findings consistent with a motor axonopathy with little or no demyelinating changes and preservation of sensory nerve potentials [49,50]. Spinal MRI may show signal abnormalities in the anterior spinal cord, consistent with anterior horn cell damage; ventral nerve root enhancement may be seen, as well (Figure 3).

Other forms of AFP, including radiculopathy and the acute demyelinating polyradiculoneuropathy form of Guillain–Barré syndrome (GBS), have also been associated with WNV infection [51,52]. However, these syndromes appear to be far less common than poliomyelitis and may be differentiated on the basis of clinical and electrophysiologic features (Table 1). The weakness associated with GBS is usually symmetric, ascending (e.g., beginning in the legs and subsequently involving the arms and cranial nerve innervated muscles) and is associated with sensory and autonomic dysfunction. Additionally, CSF examination will generally show elevated protein in the absence of pleocytosis (‘cytoalbuminologic dissociation’), and electrodiagnostic studies may be consistent with a predominantly demyelinating polyneuropathy or prominent axonal damage (in the motor axonal variant of GBS).

Recovery of limb strength in persons with WNP is variable [18,53]. However, persistent weakness and associated functional disability appears to be the rule, at least in the short term; prolonged physical and occupational therapy may be required. Most limb strength recovery occurs within the first 6–8 months after acute illness, following which improvement appears to plateau [53,54]. In particular, quadriplegia and respiratory failure are associated with high morbidity and mortality, and recovery is slow and incomplete [18]. More than 50% of the mortality associated with WNP occurs in patients with acute neuromuscular respiratory failure; of patients who survive respiratory failure due to WNP, a substantial number require prolonged tracheostomy or long-term supplemental oxygen [18,54]. In general, less profound initial weakness may be associated with more rapid and more complete strength recovery [18]. However, even patients with initially severe and profound paralysis may experience substantial recovery [18,53]; thus, as in encephalitis, the initial severity of paralysis is not necessarily a prognosticator of eventual outcome. This recovery phenomenon may in part be due to the involvement of a large number of motor neurons, which may initially be reversibly damaged, but are able to recover [42]. In addition, patients with WNP may be able, over time, to compensate for their motor deficits through the adaptation of motor skills, leading to improved functional recovery [55]. Electrodiagnostic studies may be useful in predicting the recovery of muscle strength, with subsequent improvement correlating with motor unit number estimate (MUNE) values [42]. Although case reports have suggested the occurrence of relapsing or delayed-onset cases of WNP [56], the long-term clinical and functional outcomes in patients with WNP is still emerging, and whether there may be the subsequent development of a delayed, “post-polio”-like syndrome years after acute illness is unknown. Subsequent prospective long-term evaluations of patients suffering from WNP will ultimately address this important question.

## 3. Other Clinical Manifestations of West Nile Virus Infection

### 3.1. Ocular Manifestations

Ocular manifestations, including chorioretinitis and vitritis, are the most commonly reported clinical manifestation of WNV infection after fever and neuroinvasive disease [57,58,59,60,61,62]. The optic nerve and retina are essentially extensions of the CNS, but will be considered ocular manifestations in this review. Chorioretinal lesions associated with WNV infection have been described as multifocal and with a “target-like” appearance [58]; retinal hemorrhages have also been noted. Lesions tend to be clustered primarily in the temporal and nasal regions of the periphery of the fundus. This distribution and appearance of the chorioretinal lesions have been suggested to be distinctive for WNV infection [57]. One study in Tunisia identified chorioretinitis in 20 (69%) of 29 patients with laboratory-confirmed, symptomatic WNV infection [63]; the authors concluded that ophthalmoscopic examination should be performed on all patients with suspected WNV disease. 

An inflammatory vitritis has occurred concomitantly with the chorioretinitis and may be significant enough to obscure the optic disc. Symptomatic persons describe gradual visual blurring and loss, floaters and flashes. Although experience with management is limited, improvement both in symptoms and in underlying chorioretinal lesions has been observed following treatment with intraocular corticosteroids [58]. Occasional cases of optic neuritis (inflammation of the optic nerve) have been described in the setting of WNV infection [64]). To date, WNV has not been isolated intra-orbitally.

### 3.2. Other Miscellaneous Manifestations

Numerous other clinical manifestations have been described in association with WNV infection; generally, these manifestations have been described in case reports or small case series, and a definitive causal association with WNV infection is difficult to substantiate. Rhabdomyolysis has been temporally associated with WNV infection [38,45], suggesting a viral myositis, but the presence of virus in muscle tissue has not been observed. Hepatitis and pancreatitis have been reported in cases of severe WNV infection [65,66], and WNV has been identified in hepatic and pancreatic specimens at pathology, suggesting that viscerotropic WNV disease may be an infrequent manifestation of infection. Myocarditis has been seen pathologically in WNV infection, and cardiac arrhythmias have occurred in persons with WNP, suspected to be due to autonomic dysfunction [67].

A study published in 2010 identified WNV RNA in the urine of five (20%) of 25 patients at up to seven years following acute WNV illness [68]. Four of the patients reported persistent subjective symptoms, and one patient had developed renal failure after their acute WNV illness. However, one subsequent study found no evidence of WNV RNA in urine samples collected from 40 patients at 6.5 years after acute WNV illness, and another study detected WNV RNA in the urine of only one (1.6%) of 63 persons tested <5 months after initial acute WNV infection [69,70]. The frequency of the persistence of WNV RNA in urine is unknown, and the clinical implications, if any, require further substantiation. 

## 4. West Nile Virus Long-Term Clinical and Functional Outcomes

As the clinical experience with WNV infection has extended over a period of years, our understanding of the short- and long-term outcomes of WNV infection has grown. However, detailed data regarding these long-term neurologic and functional outcomes of WNV is still relatively sparse. As with other viral encephalitides, initial severe neurologic illness does not necessarily correlate with eventual outcome, and some patients with initial severe encephalopathy with associated coma may experience good recovery and minimal sequelae [3]. However, others experience persistent neurologic dysfunction, including movement disorders, headaches, fatigue and cognitive complaints. Large hospital-based series suggest that patients with severe WNE frequently require assistance with daily activities following acute care discharge [4,5]. Patients frequently report functional and cognitive difficulties for over a year following acute infection, and only 37% of patients in the 1999 New York City outbreak achieved full recovery at one year [71]. Of 265 persons developing symptomatic WNV infection in Idaho between 2006 and 2008, 53% reported one or more persistent symptom six months or more following acute illness; the most frequent complaints were fatigue, muscle aches and difficulties with memory and concentration [72]. Cognitive complaints, including difficulties with attention and concentration, have been described among patients recovering from WNE and suggest a subcortical type of cognitive dysfunction based on prominent thalamic and basal ganglia involvement [3]. However, limited formal and objective neuropsychometric assessments have been performed. A few studies have shown that persons recovering from WNV illness demonstrate measurable neurocognitive deficits on standardized testing as long as one year after acute illness [73,74]. Other studies, however, have shown that persons recovering from WNV illness do not perform significantly differently on standardized neurocognitive assessments based upon the nature of clinical illness (e.g., WNF compared to WNM or WNE) or in comparison to unaffected persons [15]. However, self-reported fatigue, somatic and cognitive complaints are common among persons recovering from WNV illness, and subjective complaints and poorer performance on self-reported functionality indices have been seen in patients months or years following acute illness [71,75]. One study has suggested the normalization of self-reported symptoms within one year of acute illness [76]. As previously noted, neuropsychiatric symptoms, including depression and severe anxiety, have been reported by patients recovering from WNE [3,35,77]. 

## 5. West Nile Virus Infection in Children

Most children with symptomatic WNV infection present with WNF; of those who develop neuroinvasive disease, it most frequently manifests as meningitis [78,79,80]. However, severe and fatal encephalitis [81], poliomyelitis [82,83], rhombencephalitis [84] and hepatitis [85] have all been described in children with WNV infection. Similar to adults, immunocompromised children may be more susceptible to more severe illness [21]. 

## 6. Risk Factors for Severe West Nile Virus Illness and Death

Of all infected persons, less than 1% develop West Nile virus neuroinvasive disease (WNND). Although WNND has been reported among all ages, the proportion of persons who progress to WNND is greater among older compared to younger persons [8]. Serologic surveys in Romania and New York City indicate that WNV infection incidence is constant across all age groups during outbreaks [86,87], although serosurvey data are limited. Surveillance data from the United States indicate that age is the most important host risk factor for the development of WNND after infection. The incidence of neuroinvasive disease increases approximately 1.5-fold for each decade of life, resulting in a risk approximately 30 times greater for persons 80–90 years old compared to children younger than 10 years old [8]. During outbreaks, hospitalized persons older than 70 years of age had case fatality rates of 15% in Romania [86] and 29% in Israel [88]. Encephalitis with severe muscle weakness and change in the level of consciousness were also prominent clinical risk factors predicting fatal outcome [88,89]. 

Based upon a limited number of cases, patients who acquire WNV infection from infected donor organs are likely at a higher risk for severe neurologic disease and death compared with patients infected through the natural route of mosquito bite inoculation [90,91]. The risk of severe neurologic disease among other organ transplant recipients is not well-defined and may be related to the interval between infection and transplantation or the type of post-transplant immunosuppressive therapy. A seroprevalence study carried out in a Canadian outpatient transplant clinic following a WNV epidemic in 2002 indicated that the risk of neuroinvasive disease following infection was 40% (95% confidence interval 16%–80%) [92]. During that epidemic, transplant patients were approximately 40 times more likely than the general population to develop WNND [93]. However, a subsequent study assessed seropositivity and incidence of WNND among 194 solid organ transplant recipients and 195 controls and found no significant difference in seropositivity for WNV IgG between the groups and determined that the incidence of WNND among the transplant recipients was low among the seropositive transplant patients [94].

In addition to increased age and organ transplantation, hypertension, cerebrovascular disease, renal disease and diabetes have also been identified as possible risk factors for WNND, and prior immunosuppression has been associated with a fatal outcome [88,89,95,96,97,98,99]. The incidence of neuroinvasive disease and the probability of death after acquiring neuroinvasive disease are slightly higher in men than in women [8]. 

## 7. Treatment and Management

There is currently no definitive treatment for WNV infection. Prevention of infection through protection from mosquito bites is therefore critical and the single most important public health measure. In the absence of definitive antiviral treatment, management of illness due to WNV infection remains supportive. Patients with otherwise uncomplicated WNF generally do not require specific intervention, though control of headache and rehydration may sometimes be needed. However, persons with documented WN viremia and patients with WNF in which other risk factors, including older age and underlying immunosuppression, are present should be observed for progression to more severe neuroinvasive disease. Patients with severe WNM may also require pain control for severe headache, and dehydration due to associated nausea and vomiting may require hospitalization for rehydration. In patients with WNE, attention to the level of alertness and airway protection is important. While seizures and increased intracranial pressure have been infrequently reported with WNE, if present, they should be managed appropriately. 

Patients with WNP may not have concurrent meningitis or encephalitis, and thus, WNV infection may not initially be suspected. This may result in the implementation of inappropriate diagnostic procedures or treatment modalities, including anticoagulation for suspected acute stroke or muscle biopsy for suspected myopathy. WNV infection should be suspected in persons developing acute asymmetric paralysis, particularly if accompanied by other signs of infection. Patients developing early dysarthria and dysphagia are at a higher risk for subsequent acute respiratory failure [18]; for this reason, hospitalization and observation of patients with poliomyelitis is prudent, and the development of dysarthria and dysphagia should be viewed with concern. Management of poliomyelitis due to poliovirus suggests that the initiation of aggressive physical activity during the acute febrile period of illness is associated with more profound and persistent weakness [100]; in the absence of additional data, the avoidance of aggressive physical activity during the acute febrile illness or during the initial 48–72 h of weakness in WNP would be a reasonable approach, with subsequent application of physical and occupational therapy.

Viremia in humans is short-lived, and WNV is usually cleared from the system by the time of clinical presentation; this fact presents a substantial theoretical obstacle for the targeting and design of specific anti-viral therapies. The recent use of several therapeutic modalities, including antiviral agents, nucleic acid analogues, missense sequences, immunomodulating agents and angiotensin-receptor blockers, has been outside the setting of carefully controlled, randomized, blinded, placebo-controlled trials; thus, anecdotal reports of the effectiveness of these agents are unsubstantiated. However, such anecdotal reports of effectiveness and the clinical desire to provide an intervention have led to empiric use. 

The immunomodulating agent, interferon-α, while again showing *in vitro* inhibition of cytotoxicity due to WNV [101], has not been fully evaluated in animal models, and data from an open-label, non-blinded trial in the United States have not suggested a clear benefit. The use of interferon-α in the treatment of the closely related Japanese encephalitis virus suggested no benefit [102].

Animal models and anecdotal reports have suggested the efficacy of high-titer WNV-specific intravenous immune globulin (IVIG) from pooled donors (Omr-IgG-am®) [103,104,105], and humanized monoclonal WNV antibodies targeting the envelope protein of the virus (MGAWN1) [106,107,108,109]. However, animal models suggest that efficacy is greatest if these therapeutics are given prior to or very shortly after the onset of clinical illness, and attempts at human randomized clinical trials to assess the efficacy of these therapeutic agents have been unsuccessful, largely due to the challenge of enrolling a sufficient number of subjects within a likely therapeutic window. Neither of these products is licensed or available for use in the United States. As a result, the prevention of initial infection is the most critical component of any approach for the management of WNV illness. 

## 8. Conclusions

Over the past decade, the understanding of the clinical spectrum of illness, as well as the immediate and longer-term outcomes associated with human WNV infection has increased substantially. However, there are remaining clinical questions that require further elucidation. Data on the long-term neurocognitive impact on patients recovering from WNE are scant, and further information is needed to ascertain long-lasting cognitive impairment following encephalitis from WNV. The parkinsonian features associated with acute WNV illness appear in most cases to be transient and resolve over time; however, recurrent- or early-onset parkinsonism in such patients due to the senescence of dopaminergic neurons remains a hypothetical possibility. Similarly, whether patients recovering from WNP will develop recurrent limb weakness in previously affected limbs years after their acute illness, akin to ‘post-polio syndrome’ seen with poliovirus, is unknown at this point, but needs assessment. In the future, additional assessment of these and other clinical manifestations of WNV infection will be critical in aiding our understanding of the pathogenesis of WNV disease and hopefully will guide management and treatment options. 
